# A systematic review of interventions aimed at increasing physical activity in adults with chronic musculoskeletal pain—protocol

**DOI:** 10.1186/2046-4053-3-106

**Published:** 2014-09-19

**Authors:** Joanne Marley, Mark A Tully, Alison Porter-Armstrong, Brendan Bunting, John O’Hanlon, Suzanne M McDonough

**Affiliations:** 1 Centre for Health and Rehabilitation Technologies, Institute of Nursing and Health, School of Health Sciences, University of Ulster, Shore Road, Newtownabbey, Co Antrim BT37 0QB, UK; 2 Centre for Public Health, School of Medicine, Dentistry and Biomedical Science, Queens University Belfast, Room 01012, Institute of Clinical Science B, Royal Victoria Hospital, Grosvenor Road, Belfast BT12 6BA, UK; 3 UKCRC Centre of Excellence for Public Health (Northern Ireland), Institute of Clinical Science B, Royal Victoria Hospital, Grosvenor Road, Belfast BT12 6BA, UK; 4 Belfast Health and Social Care Trust, Chronic Pain Service, Belfast City Hospital, 51 Lisburn Road, Belfast BT9 7AB, UK

**Keywords:** Chronic pain, Low back pain, Osteoarthritis, Physical activity, Exercise, Randomised controlled trials

## Abstract

**Background:**

Chronic musculoskeletal pain is highly prevalent, affecting around one in five people across Europe. Osteoarthritis, low back pain, neck pain and other musculoskeletal disorders are leading causes of disability worldwide and the most common source of chronic pain. Exercise and/or physical activity interventions have the potential to address not only the pain and disability associated with chronic pain but also the increased risk of morbidity and mortality seen in this population. Although exercise and/or physical activity is widely recommended, there is currently a paucity of research that offers an evidence base upon which the development or optimisation of interventions can be based. This systematic review will investigate the components of interventions associated with changes in physical activity levels in adults with chronic musculoskeletal pain.

**Methods/Design:**

This systematic review will be reported in line with the Preferred Reporting Items for Systematic Reviews and Meta-analyses (PRISMA) guidance. Randomised and quasi-randomised controlled trials of interventions aimed at increasing physical activity in adults with chronic musculoskeletal pain will be included. Articles will be identified through a comprehensive search of the following databases: CENTRAL in the Cochrane Library, the Cochrane Database of Systematic Reviews (CDSR), MEDLINE, Embase, CINAHL, PsycINFO and AMED. Two review authors will independently screen articles retrieved from the search for eligibility, extract relevant data on methodological issues and code interventions according to the behaviour change technique taxonomy (v1) of 93 hierarchically clustered techniques. As complex healthcare interventions can be modified by a wide variety of factors, data will be summarised statistically when the data are available, are sufficiently similar and are of sufficient quality. A narrative synthesis will be completed if there is insufficient data to permit a formal meta-analysis.

**Discussion:**

This review will be of value to clinicians working in chronic pain services and to researchers involved in designing and evaluating interventions.

**Systematic review registration:**

PROSPERO reference: CRD42014010640.

## Background

Chronic pain is a complex condition that can be difficult to define; the International Association for the Study of Pain (IASP) classifies pain as ‘an unpleasant sensory and emotional experience associated with actual or potential tissue damage, or described in terms of such damage’ [1]. Chronic pain is generally considered as pain that lasts longer than 3 months or beyond the timeframe expected for healing following trauma or surgery [2]. Epidemiological studies suggest that chronic pain affects around one in five people across Europe [3,4]. Global prevalence figures vary widely, ranging from around 11% to 64% of the population in higher income countries [5]. Inconsistencies in prevalence figures are likely to reflect the different ways in which chronic pain is defined, different methods of data collection and the inherent difficulties in measuring pain. However there is a general consensus that chronic pain is highly prevalent and, with ageing populations, problems associated with chronic pain are expected to get worse [3,5,6].

Chronic pain can arise for numerous reasons; however, chronic musculoskeletal disorders are the most common cause. Osteoarthritis (OA) is reported as the leading cause of chronic pain [4] and low back pain (LBP) is the most common site of chronic pain [3,4,7]. In a report of the global burden of disease [6], LBP and neck pain were found to be among the leading causes of disability worldwide. In the European Union (EU) and European Free Trade Association (EFTA) countries, LBP is the leading cause of years lived with a disability; neck pain and other musculoskeletal disorders are also highly prevalent, ranking in the top ten causes of years lived with a disability. These figures exclude OA, which itself features in the top 20 causes of disability in every EU and EFTA country.

The costs associated with managing chronic musculoskeletal pain are difficult to establish, but conditions such as OA and LBP are considered among the most expensive to treat [7-9]. In the UK, back pain alone is estimated to cost the UK economy around £12.3 billion per year [10], a figure that would equate to around 22% of the UK’s total annual health expenditure [2]. It is estimated that across Europe chronic pain costs around €300 billion or around 1.5%–3% of the gross domestic product [3,9].

### Description of the condition

Chronic pain has a profound impact on the lives of sufferers, affecting their ability to work, maintain relationships and function in normal day to day life; around a quarter will lose their employment [3,4]. In addition to suffering from high levels of physical disability, individuals with chronic pain appear to have an increased risk for developing a range of comorbid health conditions such as depression, obesity, heart disease [11-13], cancer [14] and early mortality [13-15]. A recent study has suggested that chronic musculoskeletal pain may itself be a factor in causing cardiovascular diseases; however, as noted by the authors the causal chain remains unclear and further research is needed [16].

Despite being highly prevalent, placing a huge burden on individuals’ lives and resulting in extensive costs to the economy, chronic pain has not been afforded the same priority as many other chronic conditions. It has been suggested that this is due to chronic pain often being considered as a symptom of something rather than a specific condition in its own right [17,18].

### Description of the intervention

Physical activity has been defined as any bodily movement produced by skeletal muscles resulting in energy expenditure; it occurs across several domains including activities of daily living, occupational activities, recreation and leisure [19]. Exercise is defined as ‘a subset’ of physical activity, which tends to be structured, planned and repetitive [19,20].

There is considerable evidence to support the incorporation of exercise and/or physical activity in the management of the most common types of chronic musculoskeletal disorders. Clinical guidelines for OA [21-23], LBP [24,25], and chronic pain management [26-28] each advocate that exercise and/or being physically active should be the key treatment recommendations. Scottish Intercollegiate Guidelines Network (SIGN) guidelines [28] for the management of chronic pain endorse exercise or exercise therapy, regardless of the form of exercise, and suggest those with chronic LBP should be enabled to remain physically active to improve disability in the long term. Although exercise and/or physical activity interventions appear to have a clear role in reducing pain and disability in chronic musculoskeletal pain [21-23,25,29], additional broader health benefits may also be attained.

It is well recognised that significant health benefits can be attained by increasing physical activity levels according to government guidelines [30,31]. Pain, however, is in effect, counter-intuitive to physical activity: barriers to being active or exercising such as fear, pain and catastrophization of symptoms are well documented in this population [32-34]. It may be difficult for individuals with pain, particularly those on the severe end of the pain spectrum, to make changes to their physical activity behaviours. However, if even small changes can be achieved, evidence suggests that this will lead to health gains: a meta-analysis of physical activity and the risk of coronary heart disease found that those who were physically active at levels lower than the minimum recommended amount also had significantly lower risk of coronary heart disease [35]. Similarly, in a large nationally representative sample of adult Americans (10, 535) those who engaged in some physical activity (below recommended levels) reduced their risk of cardiovascular disease mortality and all-cause mortality [36]. Indeed, it is reported that the most significant health gains arise when those who are least fit become more physically active [37].

Enabling individuals with chronic pain to be more physically active on a regular basis is likely to be central to achieving health gains and also sustained long-term benefits from interventions. However, there is limited knowledge as to how to effectively encourage chronic pain patients to adopt a more physically active lifestyle.

### How the intervention might work

Encouraging individuals to increase and sustain changes in physical activity levels requires behavioural change: behaviour change interventions have been defined as coordinated sets of activities designed to change specified patterns of behaviour [38]. It has been suggested that the effectiveness of behaviour change interventions may relate to the behaviour change techniques that are used [39]. National Institute for Health and Care Excellence (NICE) recently reported that investigating which behaviour change techniques are effective in both initiating and sustaining behaviour change, within specific populations, is a research priority [40]. The development of a taxonomy of behaviour change techniques [41-43] has invigorated the use of a standardised language to describe intervention content. The application of behaviour change techniques has been assessed using the taxonomies in a number of physical activity behaviour change interventions [39,44-47]. Across these interventions and in line with NICE guidelines for individual level behaviour change [35], some consistent techniques appear to be associated with more effective interventions: prompt self-monitoring of behaviour, providing feedback, goal setting and social support. Similarly, a Cochrane review of adherence to exercise in chronic musculoskeletal pain found some support for the use of simple behavioural strategies such as feedback and exercise contracts [48]. The extent to which physical activity interventions within the chronic pain population utilise behaviour change techniques, and the relationship this has to outcomes has not yet been systematically explored.

### Why it is important to do this review

Physical activity has robust evidence for its effectiveness in bringing about health benefits. Although further research is warranted, it also appears to play a role in reducing pain and disability associated with chronic musculoskeletal pain [49,50]. Whilst reviews of physical activity interventions have started to emerge in an attempt to identify effective behaviour change techniques, this has not yet been explored within the chronic pain literature. Indeed, of those reviews that have been conducted, many have excluded individuals with pain [46,47] limiting extrapolation of the findings. Suffering from chronic pain raises specific challenges to engaging in physical activity, and efforts are needed to determine how interventions should be developed to address these challenges.

A Cochrane review has concluded that physical activity interventions have generally shown positive results, both in the short and medium terms, in clinical and non-clinical populations [51]. Although some research has demonstrated it is possible to increase physical activity levels in those with chronic LBP [49] or OA [50,52], an understanding of which components are effective, i.e. the ‘active ingredients’ is necessary. Identifying these components will help produce an evidence base upon which interventions can be developed.

### Aim

This systematic review will investigate the behaviour change components and outcomes of interventions aimed at increasing physical activity in adults with chronic musculoskeletal pain.

### Key objectives

The key objectives of this study are the following:

1. Determine what behaviour change techniques are used in current interventions.

2. Identify key characteristics of interventions that appear to be associated with changes in physical activity levels in adults with chronic pain.

3. Determine if particular techniques or components are associated with greater effect sizes.

## Methods/design

### Criteria for considering studies for this review

#### Types of studies

All human randomised and quasi-randomised controlled trials, published and unpublished, aimed at increasing physical activity in adults with chronic pain, arising from the axial skeleton or large peripheral joints.

#### Types of participants

We will focus on adults ≥18 years of age that have received a clinical diagnosis of chronic pain. In the absence of a clinical diagnosis, chronic pain will be defined as persistent pain lasting ≥3 months. For clarity and to exclude more distinct populations, we will focus on chronic pain arising from the axial skeleton or large peripheral joints (hip, knee and shoulder). We will exclude fibromyalgia syndrome, defined rheumatological problems such as; rheumatoid arthritis, anklosing spondylitis, etc. which may require a different management strategy. We will exclude all perioperative patients. No restrictions will be made based on gender.

#### Types of interventions

Physical activity will be considered as ‘any bodily movement produced by skeletal muscles requiring energy expenditure’ [19]. All interventions must include a measure of physical activity to be included (see ‘Types of Outcome Measures’ section below). Any intervention aimed at increasing physical activity in adults with chronic musculoskeletal pain will be eligible for inclusion, e.g. educational programmes, physical activity counselling, walking programmes, aerobic or exercise classes, self-management, lifestyle interventions, etc. We will exclude site specific rehabilitative exercise interventions (e.g. back stabilisation exercises, rotator cuff strengthening exercises, etc.) unless it is clear that the intervention also addresses physical activity, by including this as a specific outcome.

For clinical trials, one group must use or receive some form of intervention aimed at increasing physical activity. We will include any 1:1 or group based intervention, centre-based or home-based, outpatient, inpatient or community settings. We will include trials with a comparative control group and trials with multiple intervention arms (comparing different types of physical activity interventions). We will not include population or community-wide interventions (e.g. mass media campaigns, built environment, etc.).

#### Types of outcome measures

The primary outcome of interest is physical activity; studies reporting before and after measurements of physical activity levels, either as a primary or secondary outcome will be included.

#### Primary outcomes

Changes in physical activity levels measured by self-reported tools (International Physical Activity Questionnaire, recall diary, etc.) and objective measures (pedometers, actigraphy, activity monitoring global positioning systems, etc.) will be included.

#### Secondary outcome measures

A range of secondary outcomes measures are of interest and include the following:

Pain—self-reported methods such as visual analogue scale (VAS), numerical rating scale (NRS), Lequesne index or other validated assessment tool for pain.

Pain-related fear—The Pain Anxiety Symptoms Scale (PASS) or the Tampa Scale of Kinesiophobia (TSK).

Disability—back pain-specific scales (for example, the Roland-Morris Disability Questionnaire (RMDQ), or the Oswestry Disability Index (ODI)) and/or other visual analogue disability score to assess functional status/index.

Cardio-metabolic health—body mass index, blood pressure and aerobic fitness.

Quality of life and general health—health-related quality of life, e.g. SF-36 (as measured by the general health sub-scale), EuroQol, general health or similarly validated index.

Mental wellbeing—hospital anxiety and depression scale (HADS), the Beck Depression Inventory (BDI-II), State-Trait Anxiety Inventory (STAI) or similar measure of mental wellbeing.

Changes in self-efficacy—Bandura’s exercise self-efficacy scale or similar measure.

### Search methods for identification of studies

To identify studies for inclusion in this review, detailed search strategies will be developed for each electronic database searched. These will be based on the search strategy developed for Medical Literature Analysis and Retrieval System Online (MEDLINE) (Additional file 1) but revised appropriately for each database.

### Electronic searches

We will search the Cochrane Central Register of Controlled Trials (CENTRAL) in the Cochrane Library, the Cochrane Database of Systematic Reviews (CDSR) in the Cochrane Library to June 2014, Ovid MEDLINE(R) Daily Update, Ovid MEDLINE(R) 1946 to June 2014, Ovid MEDLINE®—includes new records, not yet fully indexed, Ovid Embase 1974 to June 2014, EBSCO Cumulative Index to Nursing and Allied Health Literature (CINAHL) plus 1937 to June 2014, Ovid PsycINFO 1806 to June 2014, AMED (Allied and Complementary Medicine) 1985 to June 2014.

Indexed versions of Medline will be combined with the Cochrane Search Strategy for identifying randomised trials in MEDLINE: sensitivity- and precision-maximising version (2008 revision).

CINAHL and Embase searches will be combined with the SIGN search filters developed to retrieve randomised controlled trials in these databases.

Any systematic reviews of physical activity interventions in chronic pain populations will be screened for additional references. Additional studies will be identified from the reference lists of the retrieved papers. We will supplement the electronic search strategy by using the Science Citation Index to perform citation tracking of the trials identified by the first step.

### Data collection and analysis

#### Selection of studies

Results from the searches will be imported into EndNote (X7) bibliographic software (Thomson Reuters, Philadelphia, PA, USA) and duplicates removed. The titles and abstracts of publications obtained by the search strategy will be independently screened by two authors (JM 100%, MAT 50% and SMcD 50%). Articles that do not meet the inclusion criteria will be removed. All remaining publications will be retrieved for further assessment. Based on the information within the full reports, two review authors (JM, SMcD) will use a standardised form tested prior to use to select the trials eligible for inclusion in the review, if necessary, a third review author (MAT) will resolve disagreements. A record will be kept of all articles excluded at this stage and the reason for their exclusion.

No language restrictions will be made; non-English papers will be assessed and, if necessary, translated with the assistance of a native speaker.

### Data extraction and management

Data will be extracted independently by two review authors (JM, SMcD) using a customised form, piloted prior to use. This will be used to extract relevant data on methodological issues, eligibility criteria, interventions (including the number of participants treated, intervention provider) and study design, study duration, follow-up, comparisons, outcome measures, results, withdrawals and adverse events.

Intervention content will be coded according to the behaviour change technique taxonomy (v1) of 93 hierarchically clustered techniques [42]. Two qualified coders (JM, MAT) will independently code behaviour change techniques. Kappa and percentage disagreement will be calculated, the two reviewers will meet to resolve any discrepancies, with third party adjudication if required.

In the case of multiple publications of the same study, we will, where possible, extract and combine all of the available data, in case of doubt; the original publication will be given priority. Where data seems to be missing from a study this will, if possible, be obtained through correspondence with the study authors. A table showing the characteristics of the included and excluded studies will be created.

There will be no blinding to study author, institution or journal.

### Assessment of risk of bias

Two review authors (JM, SMcD) will independently assess each included study for risk of bias using the risk of bias tool, following guidance from the Cochrane Handbook of Systematic Reviews of Interventions [53]. The following domains will be considered:

• Use of a validated measure of physical activity?

• Was the allocation sequence adequately generated?

• Was the allocation adequately concealed?

• Was knowledge of the allocated intervention adequately prevented during the study?

• Were incomplete outcome data adequately addressed?

• Are reports of the study free of suggestion of selective outcome reporting?

• Was the study apparently free of other problems that could put it at a high risk of bias?

To minimise bias in interpretation of the tool, a small sample of unrelated studies will be assessed. Inconsistency in scoring will be reviewed, and a consensus reached prior to the analysis of the review studies. The tool will be used to judge and report whether a trial is deemed to be at ‘high’, ‘low’ or ‘uncertain’ risk of bias. A summary statement regarding the quality of the data included in the review and a narrative account of any serious flaws will be reported.

### Measures of treatment effect

For each study, relative risk and 95% confidence intervals will be calculated for dichotomous outcomes, and mean differences and 95% confidence intervals will be calculated for continuous outcomes. Where continuous outcomes are pooled on different scales, standardised mean differences will be used. Where available, changes from baseline (mean change scores) will be used in preference to follow-up scores.

### Unit of analysis issues

We anticipate two possible unit of analysis issues that may arise; repeated observations of the same outcome and studies including multiple intervention arms.

We anticipate that primarily continuous data will be reported for physical activity outcomes. If studies report multiple observations of the same outcome, we will extract data at the following time points: baseline, short-term (not longer than 12 weeks post-randomisation), medium-term (not longer than 6 months post-randomisation), and long-term (greater than 6 months post-randomisation) follow-up.

In the case of studies including multiple intervention groups, we will follow the recommended method suggested by the Cochrane Collaboration section 16.5 [53] for combining multiple groups from one study. For continuous outcomes, means and standard deviations will be combined using methods and formulae described in chapter 7 (7.7.3.8) - combining groups, in the Cochrane Handbook of Systematic Reviews of Interventions.

The following will be considered in relation to assessing the risk of bias in multiple intervention studies:

• Are data presented for each of the groups to which participants were randomised?

• Are reports of the study free of suggestion of selective reporting of comparisons of intervention arms for some outcomes?

### Missing data

Attempts will be made where necessary to contact original investigators to request missing data. If standard deviations are missing from continuous data, studies will be scanned for other statistics such as confidence intervals, standard errors, or *p* values that would allow for its calculation. If there are a large number of missing standard deviations, then imputation will not be carried out.

### Assessing for heterogeneity

Diversity across the studies will be qualitatively assessed in terms of intervention (content, duration, frequency, provider and setting), participant demographics, outcome measures and follow-up. If two or more studies are considered clinically homogenous according to the above terms, data will be assessed for statistical heterogeneity using RevMan version 5.1. We will use the chi-squared (*χ*^2^) test in conjunction with the *I*^2^ statistic. The level of significance for the χ^2^ will be set at *p* < 0.1. Values of *I*^2^ that are 30% to 60% will be considered to represent moderate heterogeneity and 50% to 90% substantial heterogeneity [53]. In the case of substantial heterogeneity, we will pool studies using a random effects model; in the case of low or no heterogeneity, we will analyse studies using a fixed effects model. A sensitivity analysis will be performed to investigate the effect of inclusion and exclusion of heterogeneous studies.

### Assessment of reporting bias

A funnel plot will be prepared if there are sufficient studies by plotting trial effect against standard error. The plot will be inspected for asymmetry to investigate reporting bias.

### Data synthesis

In complex healthcare interventions, effects can be modified by a wide variety of factors and we anticipate a high degree of heterogeneity within the included studies. Careful consideration will be given to the appropriateness of conducting a meta-analysis. Data will be summarised statistically when the data is available, is sufficiently similar and of sufficient quality. The statistical analysis will be performed in accordance with the statistical guidelines referenced in version 5.1.0 of the Cochrane Handbook for Systematic Reviews of Interventions [53].

A narrative synthesis will be completed if there is insufficient data to permit a formal meta-analysis. The narrative synthesis will attempt to summarise the current state of knowledge, describe the interventions, study designs and the robustness of the evidence.

### Subgroup analysis and investigation of heterogeneity

Outcome assessment data for all time periods where available will be grouped into three time periods for the purposes of analysis: baseline (0 to 3 months), medium-term (3 to 6 months), and long-term follow-up (greater than 6 months).

Where possible, the following subgroup analysis will be performed:

• Behaviour change techniques used (previous studies have suggested particular behaviour change techniques may be associated with effectiveness).

• Duration and/or frequency of intervention (previous reviews have noted correlations between effect and duration of interventions).

• Recruitment route— i.e. primary care or specialist pain service (those accessing specialist pain services may be indicative of a more severe population).

• Clinical conditions (low back pain, osteoarthritis of hip, etc.)

### Sensitivity analysis

A sensitivity analysis may be performed to check if including or excluding studies of lower methodological rigour or higher risk of bias affects the comparison between groups. If sensitivity analysis appears to influence the findings of the review, this will be reported in the ‘Discussion’ section.

## Discussion

Physical activity interventions have the potential to address not only the pain and disability associated with chronic musculoskeletal pain but also the increased risk of morbidity and mortality seen in this population. Reviews identifying effective components of physical activity interventions have begun to emerge in clinical and non-clinical populations interventions [39,44-46], but to date, these have not been conducted within chronic pain populations. The findings of this review may be applied in clinical settings but will also be of value to those involved in designing and developing complex healthcare interventions.

## Abbreviations

IASP: International Association for the Study of Pain; OA: osteoarthritis; LBP: low back pain; EU: European Union; EFTA: European Free Trade Association; NICE: National Institute for Health and Care Excellence; SIGN: Scottish Intercollegiate Guidelines Network; VAS: visual analogue scale; NRS: numerical rating scale; PASS: The Pain Anxiety Symptoms Scale; TSK: Tampa Scale of Kinesiophobia; RMDQ: Roland-Morris Disability Questionnaire; ODI: Oswestry Disability Index; SF-36: Short form 36; HADS: hospital anxiety and depression scale; BDI-II: The Beck Depression Inventory; STAI: State-Trait Anxiety Inventory.

## Competing interests

The authors declare that they have no competing interests.

## Authors’ contributions

JM participated in the conception and design of study, developed the initial search strategy, collected background data and prepared the first draft of the manuscript. SMcD was involved in the conception and design of study, refinement of search strategy, reviewing drafts, inputting on methodology and intellectual content. MAT and APA were involved in refining search strategy, critical revisions and reviewing methodology. BB provided input on the methodology. JO’H provided input on clinical application. All authors critically reviewed the manuscript and approved the final version submitted for publication. All authors read and approved the final manuscript.

## Supplementary Material

Additional file 1 Medline search strategy. Click here for file
