# Supplementary material for: A systematic review of interventions aimed at increasing physical activity in adults with chronic musculoskeletal pain—protocol
Source: Syst Rev. 2014 Sep 19;3:106. doi: 10.1186/2046-4053-3-106 (PMC4179220; doi:10.1186/2046-4053-3-106)
Supplement: Additional file 1 — Medline search strategy. [file 2046-4053-3-106-S1.docx]

**Additional file 1**

**Search Strategy for Medline**

**[mp. = indicates searches for words in the title, abstract and MeSH descriptors]**

MEDLINED INDEXED 20.06.14

1. exp Chronic Pain/

2. chronic pain.mp.

3. 1 or 2

4. (chronic adj2 (neck or cervical or shoulder or thoracic or spin* or lumbar or low back or hip or knee or musculoskeletal) adj3 pain).mp. [mp=title, abstract, original title, name of substance word, subject heading word, keyword heading word, protocol supplementary concept word, rare disease supplementary concept word, unique identifier]

5. exp Low Back Pain/

6. low back pain.mp.

7. 5 or 6

8. lumbago.mp. [mp=title, abstract, original title, name of substance word, subject heading word, keyword heading word, protocol supplementary concept word, rare disease supplementary concept word, unique identifier]

9. Musculoskeletal Pain/

10. exp Osteoarthritis/

11. (osteoarthritis adj3 (neck or cervical or shoulder or thoracic or spin* or lumbar or low back or hip or knee)).mp. [mp=title, abstract, original title, name of substance word, subject heading word, keyword heading word, protocol supplementary concept word, rare disease supplementary concept word, unique identifier]

12. degenerative joint disease.mp.

13. Motor Activity/

14. exp Exercise/

15. exercise.mp.

16. (physical* adj1 activ*).mp. [mp=title, abstract, original title, name of substance word, subject heading word, keyword heading word, protocol supplementary concept word, rare disease supplementary concept word, unique identifier]

17. Walking/

18. walking.mp.

19. 17 or 18

20. Bicycling/

21. cycl*.mp. [mp=title, abstract, original title, name of substance word, subject heading word, keyword heading word, protocol supplementary concept word, rare disease supplementary concept word, unique identifier]

22. (aerobic adj2 (exercis* or activi* fitness)).mp. [mp=title, abstract, original title, name of substance word, subject heading word, keyword heading word, protocol supplementary concept word, rare disease supplementary concept word, unique identifier]

23. leisure activi*.mp. [mp=title, abstract, original title, name of substance word, subject heading word, keyword heading word, protocol supplementary concept word, rare disease supplementary concept word, unique identifier]

24. (physical adj1 (fitness or education or training)).mp. [mp=title, abstract, original title, name of substance word, subject heading word, keyword heading word, protocol supplementary concept word, rare disease supplementary concept word, unique identifier]

25. "Activities of Daily Living"/

26. activities of daily living.mp.

27. 25 or 26

28. pedometer.mp.

29. (self adj1 (help or managment)).mp. [mp=title, abstract, original title, name of substance word, subject heading word, keyword heading word, protocol supplementary concept word, rare disease supplementary concept word, unique identifier]

30. randomized controlled trial.pt.

31. controlled clinical trial.pt.

32. randomized.ab.

33. placebo.ab.

34. clinical trials as topic.sh.

35. randomly.ab.

36. trial.ti.

37. 30 or 31 or 32 or 33 or 34 or 35 or 36

38. exp animals/ not humans.sh.

39. 37 not 38

40. 3 or 4 or 7 or 8 or 9 or 10 or 11 or 12

41. 13 or 14 or 15 or 16 or 19 or 20 or 21 or 22 or 23 or 24 or 27 or 28 or 29

42. 40 and 41

43. 39 and 42
